# Narrowband ultraviolet B and psoralen plus ultraviolet A phototherapy treatment for mycosis fungoides: a 17-year retrospective cohort study in a university teaching hospital, 2007–24

**DOI:** 10.1093/skinhd/vzag039

**Published:** 2026-06-09

**Authors:** Thandiwe Banda, Kashini Andrew, Danning Li, Udoka Ogbuneke, Husain Alqari, Caitlin McNeil, Amirtha Rajasekaran

**Affiliations:** Birmingham Skin Centre, Birmingham City Hospital, Birmingham, UK; Birmingham Skin Centre, Birmingham City Hospital, Birmingham, UK; Birmingham Skin Centre, Birmingham City Hospital, Birmingham, UK; Birmingham Skin Centre, Birmingham City Hospital, Birmingham, UK; Birmingham Skin Centre, Birmingham City Hospital, Birmingham, UK; Birmingham Skin Centre, Birmingham City Hospital, Birmingham, UK; Birmingham Skin Centre, Birmingham City Hospital, Birmingham, UK

## Abstract

**Background:**

Mycosis fungoides (MF) is the most common type of cutaneous T-cell lymphoma. MF is typically managed with narrowband ultraviolet B (NB-UVB) or psoralen plus ultraviolet A (PUVA) phototherapy. However, comparative data on their efficacy remain limited.

**Objectives:**

To evaluate the treatment outcomes of NB-UVB and PUVA in MF and to identify clinical and demographic predictors of response.

**Methods:**

This was a retrospective cohort study of 33 patients with biopsy-proven MF treated with NB-UVB or PUVA between 2007 and 2024 in the dermatology department at the Sandwell and West Birmingham Hospitals National Health Service Trust. Statistical analysis was performed using Microsoft Excel version 2023 and R version 2023.03.1 + 446.

**Results:**

Of the 33 patients (median age 58 years), 26 (79%) received NB-UVB, 5 (15%) PUVA and 2 (6%) both. Complete remission was achieved in 15 cases (45%) and partial remission in 15 (45%), with 3 (9%) showing no response. Patients with hypopigmented MF had significantly better outcomes than those with hyperpigmented MF (*P* = 0.02). Advanced stage at treatment initiation was associated with poorer outcomes (*P* = 0.009). No significant difference was observed in response by sex, age or type of phototherapy. Phototherapy was well tolerated, with adverse effects in only three patients (9%).

**Conclusions:**

NB-UVB and PUVA are effective and well tolerated for the treatment of MF. Hypopigmented variants and ­early-disease stage predicted better outcomes.

## Teaser

This 17-year retrospective cohort study evaluated the effectiveness of narrowband ultraviolet B and psoralen plus ultraviolet A phototherapy in early-stage mycosis fungoides. Both modalities achieved high remission rates with minimal adverse effects, with hypopigmented variants and early-stage disease predicting better outcomes.

What is already known about this topic?

- Phototherapy is an effective first-line treatment for mycosis fungoides (MF).
- Narrowband ultraviolet B (NB-UVB) and psoralen plus ultraviolet A (PUVA) are well tolerated, with a low incidence of adverse effects.

What does this study add?

- NB-UVB and PUVA are equally effective for the treatment of MF, with no significant difference in remission rates.
- Hypopigmented MF and early disease stage predict better outcomes.

Mycosis fungoides (MF) is the most common subtype of primary cutaneous T-cell lymphoma (CTCL), and it is often diagnosed in its early stages (IA–IIB). Together with Sézary syndrome, another form of CTCL, they account for approximately 50–60% of all cases of primary CTCL. MF has an annual incidence of 0.7 per 100 000 in the UK and typically occurs in individuals during their sixth decade of life, with a higher prevalence in men (male-to-female ratio 1.5–2: 1).^1,2^

MF is characterized by malignant T-cell infiltration of the skin, with an initial clinical presentation as patches or plaques that may progress to tumours or erythroderma. Diagnosis is challenging and delayed, often requiring multiple skin biopsies. The early lesions of MF typically present as eczematous patches with well-defined borders, predominantly affecting non-sun-exposed areas. Diagnosis is often delayed due to the subtle nature of these lesions, further complicated by various clinical variants, including poikilodermatous, hypopigmented, hyperpigmented, pustular or papular, lichenoid and bullous forms.^3^

At present, there is no cure for MF, except for allogenic stem cell transplantation, which is the only treatment with curative potential. Due to the chronic and often progressive nature of MF, treatment strategies focus on symptom control, delaying disease progression and improving patients’ quality of life.^4,5^

The primary treatment for early-stage MF mainly involves topical skin-directed therapies such as high-potency topical corticosteroids, and topical chemotherapy such as mechlorethamine. Other early-stage therapies include phototherapy, total-skin electron beam therapy and radiotherapy for tumours.^2^ Systemic treatments are considered when skin-directed therapies prove insufficient. Superpotent topical steroids are a very effective first-line treatment for patches and some plaques; however, effectiveness is short-lived and recurrence is frequent for early-stage disease. Topical chlormethine, another first-line treatment for CTCL, is effective in just over 50% of cases and works by destroying DNA, inhibiting plaque growth and enhancing the immune response.^3,6^

Phototherapy, which utilizes ultraviolet (UV) radiation, remains a cornerstone in MF management. Among available options, ­narrowband UVB (NB-UVB) and psoralen plus UVA (PUVA) have demonstrated efficacy in inducing remission in cutaneous lesions via their cytotoxic and antiproliferative effects.^7^ NB-UVB emits a narrow wavelength spectrum (311–313 nm) and is particularly effective for superficial skin lesions, while PUVA combines UVA exposure with a photosensitizing agent, psoralen, enabling deeper skin penetration and enhanced therapeutic effects.^7^ Despite their widespread use, data on the comparative effectiveness of these modalities in different patient demographics and disease stages remain limited.

The study evaluated overall treatment response, compared response rates across disease stages, and examined the correlation between total phototherapy dose and outcomes. It also explored the influence of demographic factors, including age and sex, on treatment efficacy.

## Patients and methods

We conducted a single-centre, longitudinal, retrospective analy­sis of 33 patients with MF treated with either NB-UVB or PUVA phototherapy at Sandwell and West Birmingham Hospitals Trust, Birmingham, UK. Patients were identified using the hospital electronic records. The inclusion criteria were patients with ­biopsy-proven MF stage IA–IIB treated with NB-UVB or PUVA between January 2007 and July 2024. Patients were excluded if they had stopped phototherapy or were lost to follow-up before a response to treatment was documented.

Staging was classified using the TNM classification of the International Society for Cutaneous Lymphoma and Cutaneous Lymphoma Task Force of the European Organisation for Research and Treatment of Cancer.^7^ Response to treatment was defined as complete response (100% clearance of skin lesions), partial response (50–99% clearance) or no response (<50% clearance).^8^ Relapse was defined as disease recurrence in those with complete or partial response.^8^ Duration of remission was defined as the period between complete response and relapse.

### Narrowband ultraviolet B

Patients were treated with NB-UVB phototherapy three times a week with a minimum 48-h interval between treatments. The usual course of treatment was 20–30 doses depending on efficacy. The starting dose was dependent on skin type and the dose was increased by 20% at each treatment visit. If the previous dose resulted in grade E1 erythema the dose was repeated and then the next dose increment was reduced to 10%. If the previous dose caused type E2 erythema the next dose was 50% of the previous dose and then the dose was increased by 10% increments. If the patient developed E3 or E4 erythema, treatment was suspended.^9^

### Psoralen plus ultraviolet A

Patients ingested 8-methoxypsoralen, dosed according to estimated body surface area, between 0.4 and 0.6 mg kg^−1^ bodyweight. This was ingested 2 h prior to UVA therapy. Patients were treated with PUVA twice weekly with a minimum 72-h interval between treatments. The usual course of treatment was 20–30 sessions, with a maximum of 30 sessions; the maximum dose per treatment was 12 J cm^−2^.

The starting dose was dependent on skin type and the dose was increased by 20% each session. In the case of grade E1 erythema, the previous dose was repeated and the dosage changed by a 10% increment each dose. If the previous dose caused E2 erythema, the next treatment was postponed, and the previous dose was repeated at the next visit and increments reduced to 10%, with a further reduction to 5%. If the previous dose caused E3 erythema, the next dose was 50% of the previous dose and then the dose was increased by 10% increments. If the treatment caused E4 erythema, treatment was suspended.

### Choice of treatment

NB-UVB was used as first-line phototherapy for patients with ­early-stage MF, particularly those with predominantly patch-stage disease or thinner plaques, in line with national UK phototherapy guidelines.^9^ PUVA was reserved for patients with thicker plaques or for those who demonstrated an inadequate clinical response to NB-UVB. Patients who received both modalities were initially treated with NB-UVB and subsequently escalated to PUVA within the same treatment course due to insufficient clinical response.

### Data extraction and analysis

Sixty patients were identified as having MF and received phototherapy treatment. Twenty-seven patients were excluded due to missing data. Thirty-three patients were included in the final analyses. Medical records were reviewed to collect demographic data, including age at time of treatment, sex, disease stage and clinical variant of MF (hyper- or hypopigmented). Treatment data were collected, including the cumulative dose of phototherapy, stage of MF, response to treatment and duration of remission.

### Statistics

The nature of the modelling and data handling meant it was not possible to blind either the authors or statisticians to the outcome. Independent variables for treatment outcome were defined as age, sex, type of phototherapy used (PUVA or NB-UVB), number of treatment sessions and pre-existing disease severity. Normality testing was performed with the Shapiro–Wilk test. Age distribution was parametric, and the data distributions of all of the other continuous variables were nonparametric (Table S1; see Supporting Information). The dependent variable was defined as failure, partial clearance or complete clearance of disease.

For all analyses, *P*-values <0.05 were considered statistically significant. Data were analysed using Microsoft Excel version 2023 and R version 2023.03.1 + 446 (R Foundation for Statistical Computing, Vienna, Austria). Missing data were treated as missing. The results were rounded to three significant figures.

## Results

### Patient demographics

The study included 33 patients with a median age of 58 years [interquartile range (IQR) 34.5–75, range 15–96]. The median number of treatment sessions for NB-UVB or PUVA was 34.5 (IQR 28–40, range 19–121). The median cumulative dose of phototherapy was 72.3 J cm^−2^ (IQR 38.0–106, range 1.9–2235). Among the participants, 16 (48%) were men and 17 (52%) were women (Table 1). The hyperpigmented MF type was observed in 13 (39%) patients and the hypopigmented type in 17 (52%), while in 3 (9%) patients the type was not documented. The majority of patients (*n* = 21, 64%) had stage IA MF, nine (27%) had stage IB MF, one (3%) had stage IIA MF and two (6%) had stage IIb MF. Most patients received NB-UVB therapy (*n* = 26; 79%), while five (15%) underwent PUVA therapy. Two patients (6%) received sequential NB-UVB and PUVA therapy. These patients were initially treated with NB-UVB but were transitioned to PUVA within the same treatment course due to inadequate clinical response. Complete response was achieved in 15 (45%) patients, while 15 (45%) showed a partial response. Treatment failure was observed in three (9%) patients.

**Table 1:** Demographics of the cohort of 33 patients with mycosis fungoides

Demographic	*n* (%)^a^
Age (years), median (IQR), range	58 (34.5–75), 15–96
Number of treatment sessions, median (IQR), range	34.5 (28–40), 19–121
Cumulative dose (J cm^−2^), median (IQR), range	72.3 (38.0–106), 1.9–2235
Sex	
Male	16 (48)
Female	17 (52)
Mycosis fungoides type	
Hyperpigmented	13 (39)
Hypopigmented	17 (52)
Not documented	3 (9)
Disease stage	
IA	21 (64)
IB	9 (27)
IIA	1 (3)
IIB	2 (6)
Phototherapy type	
NB-UVB	26 (79)
PUVA	5 (15)
NB-UVB and PUVA	2 (6)
Outcome	
Complete response	15 (45)
Partial response	15 (45)
Treatment failure	3 (9)
Side effects	
No	30 (91)
Yes	3 (9)
Relapse^b^	
No relapse	8 (42)
Relapse	11 (58)

IQR, interquartile range; NB-UVB, narrowband ultraviolet B; PUVA, psoralen plus ultraviolet A. ^a^Data are presented as *n* (%) unless otherwise indicated. ^b^In 19 patients with data.

Side effects were reported in three (9%) patients, while 30 (91%) experienced no adverse effects. Reported side effects included acute urticaria, hyperpigmentation, eczematous and post­inflammatory changes, erythema, leg soreness and swelling, and a radiation burn secondary to accidental removal of protective eyewear.

### Statistical analysis

Ordinal logistic regression was performed to assess the association between age, number of sessions and cumulative dose with the ranked outcome categories (failure < partial response < complete response). The number of sessions showed a marginally significant negative association with outcome (β = −0.03, *P* = 0.05; Table 2). This was predominantly driven by the threshold for transitioning from failure to partial response (*P* < 0.001), whereas no significant negative association was found with outcome when transitioning from partial to complete response. There was no overall correlation between age and treatment outcome (β = −0.026, *P* = 0.09).

**Table 2:** Associations between outcomes and response

	β-value	SE of β-value	*t*-value	*P*-value
Age	−0.026	0.016	−1.68	0.09
F|P	−3.87	1.15	−3.35	**<0.001**
P|C	−1.23	0.91	−1.34	0.18
Number	−0.030	0.016	−1.95	**0**.**05**
F|P	−3.71	1.09	−3.41	**<0.001**
P|C	−0.92	0.71	−1.3	0.19
Cumulative doses	−0.0007	0.0008	−0.83	0.41
F|P	−2.29	0.68	−3.38	**<0.001**
P|C	0.39	0.43	0.90	0.37

Significant *P*-values are shown in bold; C, complete response; F, treatment failure; P, partial response.

Patients with hypopigmented MF had significantly better outcomes than those with the hyperpigmented subtype [*P* = 0.02, Fisher’s exact test (Table S2; see Supporting Information)]. Later-stage disease at the initiation of treatment was associated with a worse outcome (rho = 0.39, *P* = 0.009; Spearman’s rank correlation). No statistically significant difference in treatment outcomes was observed between PUVA and NB-UVB therapies or between sexes (Kruskal–Wallis test).

The likelihood of relapse among patients who achieved either partial or complete clearance was evaluated using Fisher’s exact test, yielding a *P*-value of 0.35. Relapse outcomes were documented for 19 patients, of whom 11 experienced a relapse and 8 did not.

## Discussion

This study demonstrates that PUVA and NB-UVB phototherapy are effective treatments for MF, with the majority of patients (96%) achieving complete or partial remission. There was no significant difference in remission rates between PUVA and NB-UVB phototherapy in this study.

This was also demonstrated by a meta-analysis, which found that NB-UVB and PUVA were both effective in inducing complete and partial remission in most cases, and there was no statistically significant difference between the two treatments.^10^ However, that study did note that PUVA demonstrated a superior complete response for those with stage IA and IB disease.^10^ It is also noted in the literature that those treated with PUVA may have longer remission durations, although this is supported by only a limited number of studies.^7,11^

Furthermore, some studies have found that thicker plaque MF has a better response with PUVA than NB-UVB and it is therefore recommended in the UK national phototherapy guidelines to offer PUVA for those with thicker plaques.^9^ This finding was not noted in our study, likely due to the small number of patients in the PUVA group.

Most patients had MF of disease stage IA or IB (Table 1). Later disease stage at presentation was associated with significantly worse outcomes. This suggests that patients with a later disease stage may benefit from the addition of systemic therapies such as bexarotene, interferon-α or immunotherapy. However, there is a lack of studies on combinations of systemic and skin-­directed therapies and therefore systemic therapies are not recommended as first-line treatment.^7^ Similar studies, which included only patients with stage IA and IB disease, have not found any difference in outcome by disease stage.^12–14^

A meta-analysis that included patients with stage IA, IB and IIA MF found that there were significantly fewer failed responses for NB-UVB and PUVA in those with stage IA and stage IB MF compared with stage IIA disease.^10^ Our study included patients with stage IIA and IIB disease, whereas phototherapy is typically recommended for those with plaque and patch-stage MF in the UK.^9^ Conclusions with regard to the treatment of advanced-stage MF are limited by the small cohort size in this study.

Regarding the clinical variant of MF, our study demonstrated a significantly better response in patients with the hypopigmented variant of MF, which is more common in younger patients and patients with more richly pigmented skin, in keeping with the literature.^15^ The underlying mechanism is not fully understood, but it has been suggested that the predominance of CD8^+^ cytotoxic T cells could result in a better antitumour immune response, which could potentially enhance the effects of treatments such as phototherapy.^16^ A few studies have noted that PUVA is more effective than NB-UVB for patients with hypopigmented MF; however, we did not observe this in our study, probably due to the small sample size.^16,17^ Our study also demonstrated that there was no difference in outcomes with age or sex, and the response to phototherapy was similar to that in previous studies.^12–14,18^

There was a marginally significant negative association with the number of treatment sessions and treatment outcomes. This may suggest that patients who require prolonged treatment may have more refractory disease, which may necessitate adjunct or alternative therapies.

In this study, the number of phototherapy sessions varied widely, reflecting differences in disease response and the length of the treatment course. One patient received a total of 121 phototherapy sessions, substantially higher than the rest of the cohort. This was due to multiple courses of PUVA undertaken for recurrent disease. This outlier contributed to the wide upper range in session numbers but did not materially affect the overall analysis.

Phototherapy was well tolerated, with only 9% of patients experiencing adverse effects. This highlights that phototherapy is a safe treatment for early-stage MF. An earlier meta-analysis suggested no difference in adverse effects of erythema, nausea, pruritus, phototoxic effects, dyspepsia and pain with NB-UVB in comparison with PUVA.^10^ One recent small study has demonstrated that patients with MF treated with PUVA may have a high risk of developing skin cancers. Therefore, clinicians should weigh up the benefits against the potential risks and ensure regular skin examinations and consider long-term surveillance.^19^

One of the key limitations of this study is the presence of confounding factors that might have influenced the results. Specifically, the group that experienced treatment failure included a statistically significant proportion of older patients (Table 2), and individuals with more advanced disease stages (Table S3; see Supporting Information). Due to the relatively small number of patients in each group, particularly among those in whom treatment failed, it was not feasible to perform propensity-­matched analyses to control for these confounding variables. This limitation restricts our ability to draw definitive conclusions about the relationship between treatment and outcomes, highlighting the need for further research with larger, more balanced cohorts to validate these findings. Due to the retrospective nature of the study, there are potential biases caused by missing data and incomplete documentation of treatment response. Future studies should focus on prospective, multicentre studies with larger samples sizes to validate our findings.

In conclusion, this study demonstrated that both PUVA and NB-UVB phototherapy are effective treatments for MF, with minimal adverse events reported. The authors also noted that earlier disease stage and the hypopigmented MF variant demonstrated better outcomes than the hyperpigmented variant. Future work could consider the efficacy of phototherapy as an adjunct in those with advanced-stage disease.

## Supplementary Material

vzag039_Supplementary_Data
