# Supplementary material for: Narrowband ultraviolet B and psoralen plus ultraviolet A phototherapy treatment for mycosis fungoides: a 17-year retrospective cohort study in a university teaching hospital, 2007–24
Source: Skin Health Dis. 2026 Jun 9;6(4):454–8. doi: 10.1093/skinhd/vzag039 (PMC13425031; doi:10.1093/skinhd/vzag039)
Supplement: vzag039_Supplementary_Data [file vzag039_supplementary_data.docx]

**Table S1** Normality testing

| Variable | W Statistic | p-value |
| --- | --- | --- |
| Age | 0.95957 | **0.2513** |
| Number Sessions | 0.74304 | 7.04E-06 |
| Cumulative dose | 0.42131 | 2.92E-09 |

**Table S2** Additional statistical tests

| Variable | Test | Result |
| --- | --- | --- |
| MF type (hypo or hyperpigmented) | Fisher’s exact | p = 0.0158 |
| Relapse | Fisher’s exact | p = 0.347 |
| MF stage (1a, 1b, 2a, 2b) | Spearman’s Rank | rho = 0.391, p = 0.00933 |
| Sex | Kruskal-Wallis | chi-squared = 0.359, df = 1, p = 0.549 |
| Phototherapy Type | Kruskal-Wallis | chi-squared = 2.66, df = 1, p = 0.103 |

**Table S3** Staging of patients with mycosis fungoides and ultimate outcome, P = partial response, F = failure of response, C = complete response

| Stage | Outcome |
| --- | --- |
| IA | P |
| IB | P |
| IB | P |
| IIB | P |
| IA | P |
| IB | P |
| IA | P |
| IA | P |
| IB | P |
| IA | P |
| IA | P |
| IA | P |
| IB | P |
| IA | P |
| IA | P |
| IIB | F |
| IIA | F |
| IB | F |
| IA | C |
| IA | C |
| IB | C |
| IA | C |
| IA | C |
| IB | C |
| IA | C |
| IA | C |
| IA | C |
| IA | C |
| IA | C |
| IA | C |
| IA | C |
| IA | C |
| IB | C |
